# Rage and Ruin

**Published:** 1854-06

**Authors:** 


### RAGE AND RUIN.

Some one has said, that every furious burst of passion shortens a man's life a year. If it only shortened his own life, the world would not be a great loser ; but unfortunately, passionate people keep all around them in hot water ; their very presence, without a word being said, generates an evil atmosphere, causing an apprehensive uneasiness, which annihilates every gladsome feeling. "Mother," said a little child one day, "if I am a good little girl, will I go to heaven when I die?"

"Yes, my child; and all good people go there too."

"Mother, will grandfather go to heaven when he dies?"

"Yes, my dear, I hope he will."

"Well, mother, I don't want to go to heaven, grandfather is so cross."

The Editor confesses to a *combined feeling*, a half and half mixture of sadness and impatience, whenever he loses a "case," although it is said, that the "doctor's bill" is paid more cheerfully than any other, when divers items, "real, personal and mixed," thereby change owners. But absorbed in such a feeling as above described, it is rather up hill work to sustain a cheerful countenance at the evening reunion of wifey and children, and grandmother. Tidy as the tea table may be, brightly as the "Liverpool" burns in the grate, joyfully as little Bob flaps his hands and arms, as if they were a pair of wings, the moment "father" enters the door; still, there is the incubus of the "lost patient." Sadness, that human power is so limited; impatience, that physic had not had some unusual efficacy in *this* case, because there is always *something* to constitute a particular reason for the restoration of the case in hand; the only sister in a family of loving brothers, it may be; a father, on whose constant labors depends the support of a helpless young family; a son, the hope of a widowed mother, her only stay in life; a husband, far on in years, every child gone long before him, the only solace of her heart, with whom he had lived lovingly from the day they both pledged themselves to love, and "none other" for life, yet, old as he is, and carefully as he has to be watched over, she, whose attachment, scores of years have only deepened and purified, she too feels a special desire that he might be spared to travel with her a little further, towards the borders of the "promised land," else, if he passes away, who is there in the wild, rude world, to be interested in her welfare, to look to her interests, to sympathise in her sorrows, to shed one tear at her grave?—not one! How terrible to be left alone, old, childless! When that hour comes to me, let it be my last.

But the memory of the "lost patient"—the impress it leaves on the countenance, even amid family joys, has a contaminating effect, and little Moll, our four year old, a perfect Mimosa, whose joyously beaming face will be saddened over in a second, by half a father's frown, sidles up to mother, and hiding her face in her lap, most earnestly inquires, "Mother, does thee think father loves us any?"

The Editor has been trying very hard for the last half hour to come to the point in an easy, gradual, graceful way—but "copy" is wanted—and he must close with the statement of a fact, which illustrates the point he has been aiming at, the influence it has on the health and happiness of all, especially in family circles, to strive after, and maintain an habitually cheerful quietude of deportment, at home and abroad, on the street, by the wayside; thus striving resolutely, bravely, against whatever odds, of a naturally hasty temper, so terrible an experience as the following may never fall to the lot of the reader.

A farmer, not long since, in Wanapace, Wisconsin, sold a yoke of oxen to an individual in the neighborhood, and received his pay in paper money. The man who purchased the oxen, being in a hurry to start off, requested the farmer to assist in yoking them up. He accordingly went to the yard with the man for that purpose, leaving the money lying on the table. On his return to the house, he found his little child had taken the money from the table, and was in the act of kindling the fire in the stove with it. From the impulse of the moment he hit the child a slap on the top of the head, so hard as to knock it over, and in the fall it struck its head against the stove with such force as to break its skull.

The mother, who was in the act of washing a small child in a tub of water, in an adjoining room, on hearing the fracas, dropped the child and ran to the room whence the noise proceeded, and was so much terrified at what she there beheld, that she forgot the little child in the tub for a time, and upon her return to the room found the little one drowned. The husband, after a few moments reviewing the scene before him, seeing two of his own children dead, without further reflection, took down his gun and blew his own brains out.

---

If you act with a view to praise only, you deserve none.

